# Supplementary material for: Mechanistic analysis of Riboswitch Ligand interactions provides insights into pharmacological control over gene expression
Source: Nat Commun. 2024 Sep 17;15:8173. doi: 10.1038/s41467-024-52235-3 (PMC11408619; doi:10.1038/s41467-024-52235-3)
Supplement: Supplementary file 1 — Supplementary Information [file 41467_2024_52235_MOESM1_ESM.pdf]

Mechanistic Analysis of Riboswitch Ligand Interactions Provides Insights into Pharmacological Control over Gene Expression

Shaifaly Parmar[1], Desta Doro Bume[1], Colleen M. Connelly[1], Robert E. Boer[1], Peri R. Prestwood[1], Zhen Wang[2], Henning Labuhn[2], Krishshanthi Sinnadurai[2], Adeline Feri[2], Jimmy Ouellet[2], Philip Homan[3,4] Tomoyuki Numata[5], John S. Schneekloth Jr.*[1]

[1] Chemical Biology Laboratory, Center for Cancer Research, National Cancer Institute, Frederick, MD 21702-1201, USA.

[2] Depixus, 3-5 Impasse Reille, 75014 Paris, France

[3] Center for Cancer Research Collaborative Bioinformatics Resource, National Cancer Institute, National Institutes of Health, Bethesda, MD, 20892, USA

[4]Advanced Biomedical Computational Science, Frederick National Laboratory for Cancer Research, Frederick, MD, 21702, USA

[5] Department of Bioscience and Biotechnology, Graduate School of Bioresource and Bioenvironmental Sciences, Kyushu University, Fukuoka 819-0395, Japan.

*e-mail: schneeklothjs@mail.nih.gov

Supplementary Information

Table S1: List of riboswitch constructs

	T7 promoter-**aptamer sequence**-RT primer binding site
B. subtilis	*TAATACGACTCACTATAG*GGCCTTCGGGCCAAGCGGG**AGAGGTTCTAGCTACACCCTCTATAAAAAACTAA**GGACGAGCTGTATCCTTGGATACGGCCTTTTTCGATCCGGTTCGCCGGATCCAAATcgggcttcggtccggttc
T. tencongensis	*TAATACGACTCACTATAG*GGCCTTCGGGCCAACTCAC**CTGGGTCGCAGTAACCCCAGTTAACAAAACA**AGGGAGGTAATTTTTCGATCCGGTTCGCCGGATCCAAATcgggcttcggtccggttc

S. saprophyticus	*TAATACGACTCACTATAG*GGCCTTCGGGCCAATATAA**AGAGGTTCCTAGCTGATACCCTCTATAAAAAACTA**GACACATGTACAACGTCTGTCTTTTTTATAGAGATAGGCGTTTTTTCGATCCGGTTCGCCGGATCCAAATcgggcttcggtccggttc
L. rhamnosus	*TAATACGACTCACTATAG*GGCCTTCGGGCCAA**GGAACCGCCCCGCGCGCTTCCACGATACTTATTTCCTTTGATCGTCGTTATTACTGGCAAAGCCACA**AAGGAGTCGATCCGGTTCGCCGGATCCAAATcgggcttcggtccggttc
F. prausnitzii	*TAATACGACTCACTATAG*GGCCTTCGGGCCAAGGAACGCTGAAA**GAGCAACTTAGGATTTTAGGCTCCCCGGCGTGTCTCGAACCATGCCGGGCCAAACCCATAGGGCTGGCGGTCCCTGTGCGGTCAAAATTCATCCGCCGGAG**TATTGTTATCGATCCGGTTCGCCGGATCCAAATcgggcttcggtccggttc
S. pneumoniae	*TAATACGACTCACTATAG*GGCCTTCGGGCCAA**CTTGGTGCTTAGCTTCTTTCACCAAGCATATTACACGCGGATAACCGCCAAAGGAGAA**TCGATCCGGTTCGCCGGATCCAAATcgggcttcggtccggttc

Table S2: Primer sequences

SHAPE_temp_F<br>SHAPE_temp_R	TAATACGACTCACTATAGGGCC<br>GAACCGGACCGAAGCC
SHAPE_PCR1_*Bsu*:	GACTGGAGTTCAGACGTGTGCTCTTCCGATCTNNNNNGCGGGAGAGGTTCTAGCTAC
SHAPE_PCR1_*Tte*:	GACTGGAGTTCAGACGTGTGCTCTTCCGATCTNNNNNCTCACCTGGGTCGCAGTAAC
SHAPE_PCR1_*Ssa*:	GACTGGAGTTCAGACGTGTGCTCTTCCGATCTNNNNNTATAAAGAGGTTCCTAGCTG
SHAPE_PCR1_*Lrh*:	GACTGGAGTTCAGACGTGTGCTCTTCCGATCTNNNNNGGAACCGCCCCGCGCGCTTC
SHAPE_PCR1_*Fpn*:	GACTGGAGTTCAGACGTGTGCTCTTCCGATCTNNNNNGGAACGCTGAAAGAGCAACT
SHAPE_PCR1_*Spn*:	GACTGGAGTTCAGACGTGTGCTCTTCCGATCTNNNNNCTTGGTGCTTAGCTTCTTTC

Reverse primer :	CCCTACACGACGCTCTTCCGATCTNNNNNGAACCGGACCGAA GCCCG

Table S3: List of oligos used in the MSF analysis

Bsu PreQ$_1$ RNA	5'biotin- UCGGCGAUCUACGCAGCGACAUAAUAAGAGGUUCUAGCUAC ACCCUCUAUAAAAAACUAAUAACAACCACUUCCUAAUCUGUCA UCUUCUG
5' DNA splint	GTCGCTGCGTAGATCGCCGA
3' DNA splint	GTGTCTTTTGGTCTTTCTGGTGCTCTTCGAATCAGAAGATGACA GATTAGGAAGTGG

Figure S1. Affinity measurements of synthetic ligands to *Bsu*-PreQ$_1$ riboswitch. Inherent ligand fluorescence of compounds **1** (A), **2** (B), **4** (D), and **8** (H) measured under increasing concentrations. MST of Cy5-labeled RNA with increasing concentrations of compounds **3** (C), **5** (E), **6** (F), **7** (G), **9** (I). Error bars indicate the standard deviation determined from three independent measurements.

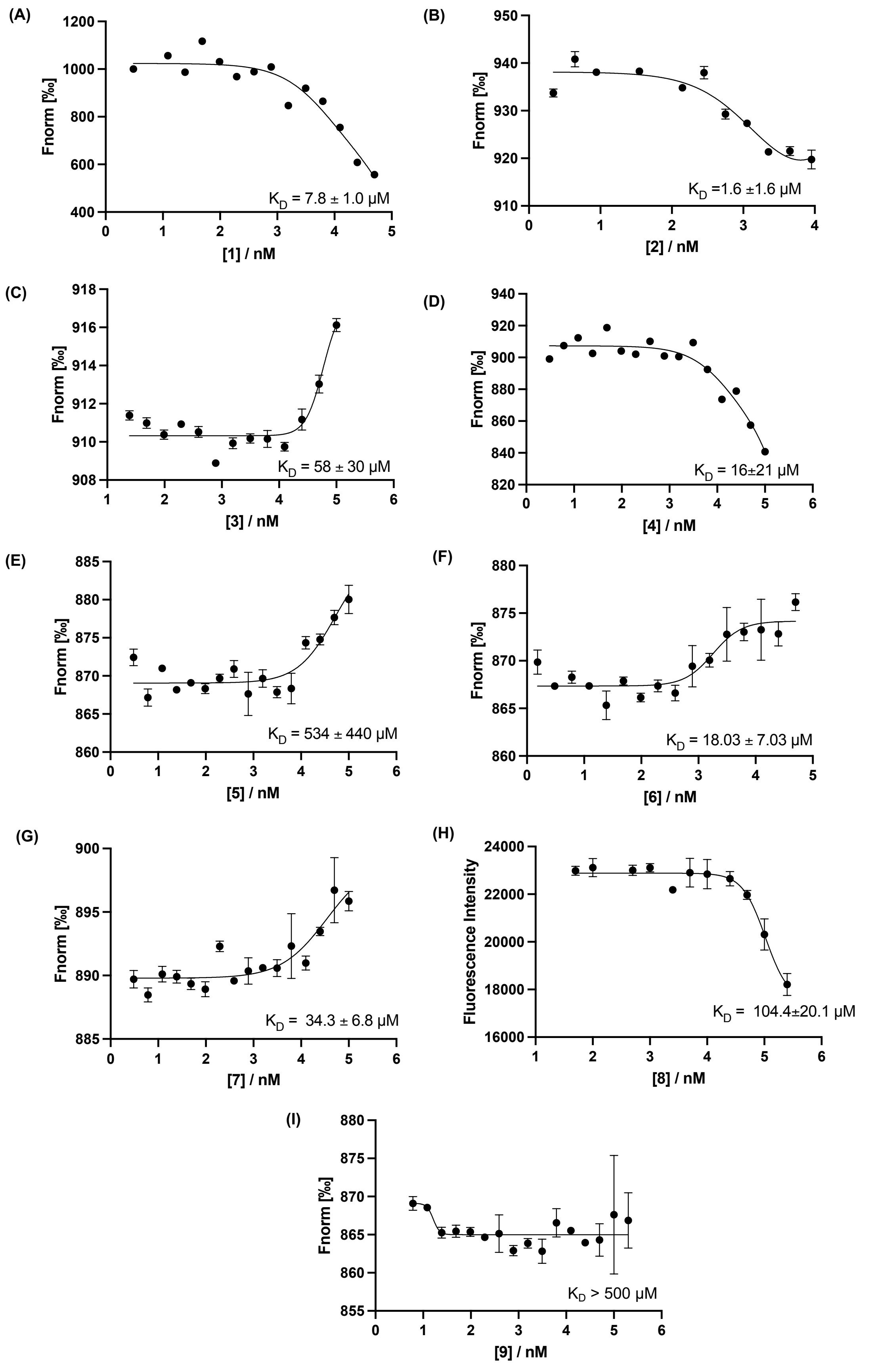
(A)
Fnorm [‰]
K$_D$ = 7.8 ± 1.0 µM
[1] / nM
(B)
Fnorm [‰]
K$_D$ =1.6 ±1.6 µM
[2] / nM
(C)
Fnorm [‰]
K$_D$ = 58 ± 30 µM
[3] / nM
(D)
Fnorm [‰]
K$_D$ = 16±21 µM
[4] / nM
(E)
Fnorm [‰]
K$_D$ = 534 ± 440 µM
[5] / nM
(F)
Fnorm [‰]
K$_D$ = 18.03 ± 7.03 µM
[6] / nM
(G)
Fnorm [‰]
K$_D$ = 34.3 ± 6.8 µM
[7] / nM
(H)
Fluorescence Intensity
K$_D$ = 104.4±20.1 µM
[8] / nM
(I)
Fnorm [‰]
K$_D$ > 500 µM
[9] / nM

Figure S2. Affinity of **4** for $PreQ_1$ aptamers. Fluorescence intensity assay of 5′-AlexaFluor 647 labeled (A) *Ssa*-$PreQ_1$ and (B) *Tte*-$PreQ_1$ riboswitch aptamers in the presence of increasing concentration of **4**. Error bars indicate the standard deviation determined from three independent measurements.

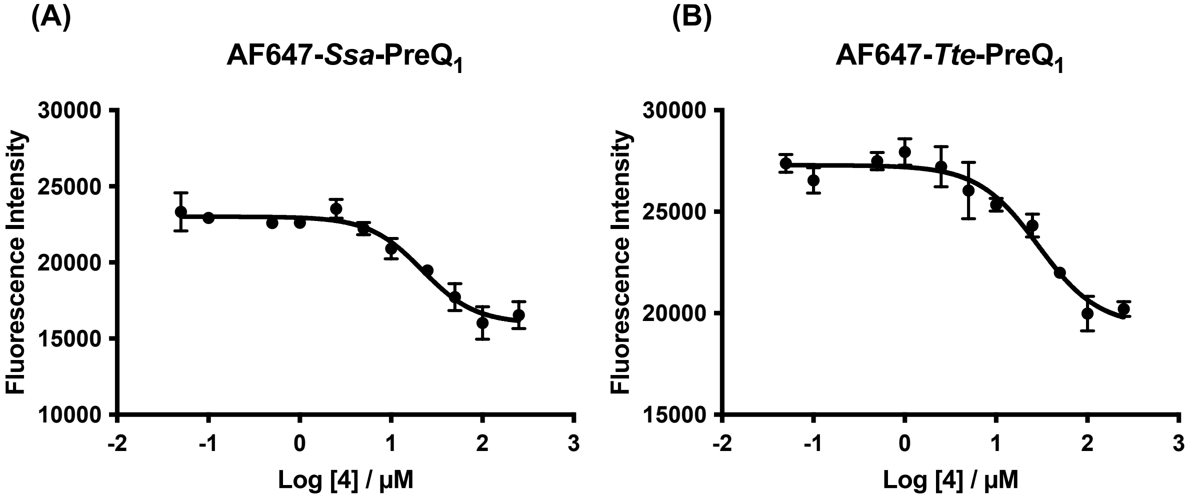

Figure S3. Specificity of **4** towards different RNA secondary structures. Affinity measurements with increasing concentrations **4** to DHX15, an RNA-G4 (A), mango II aptamer – RNA G4 (B), MYCN, a DNA-G4 (C) SARS-CoV2 pseudoknot (D), SARS-CoV2-FSE stem loop (E), U4U6 3-way junction (F). Error bars indicate the standard deviation determined from three independent measurements.

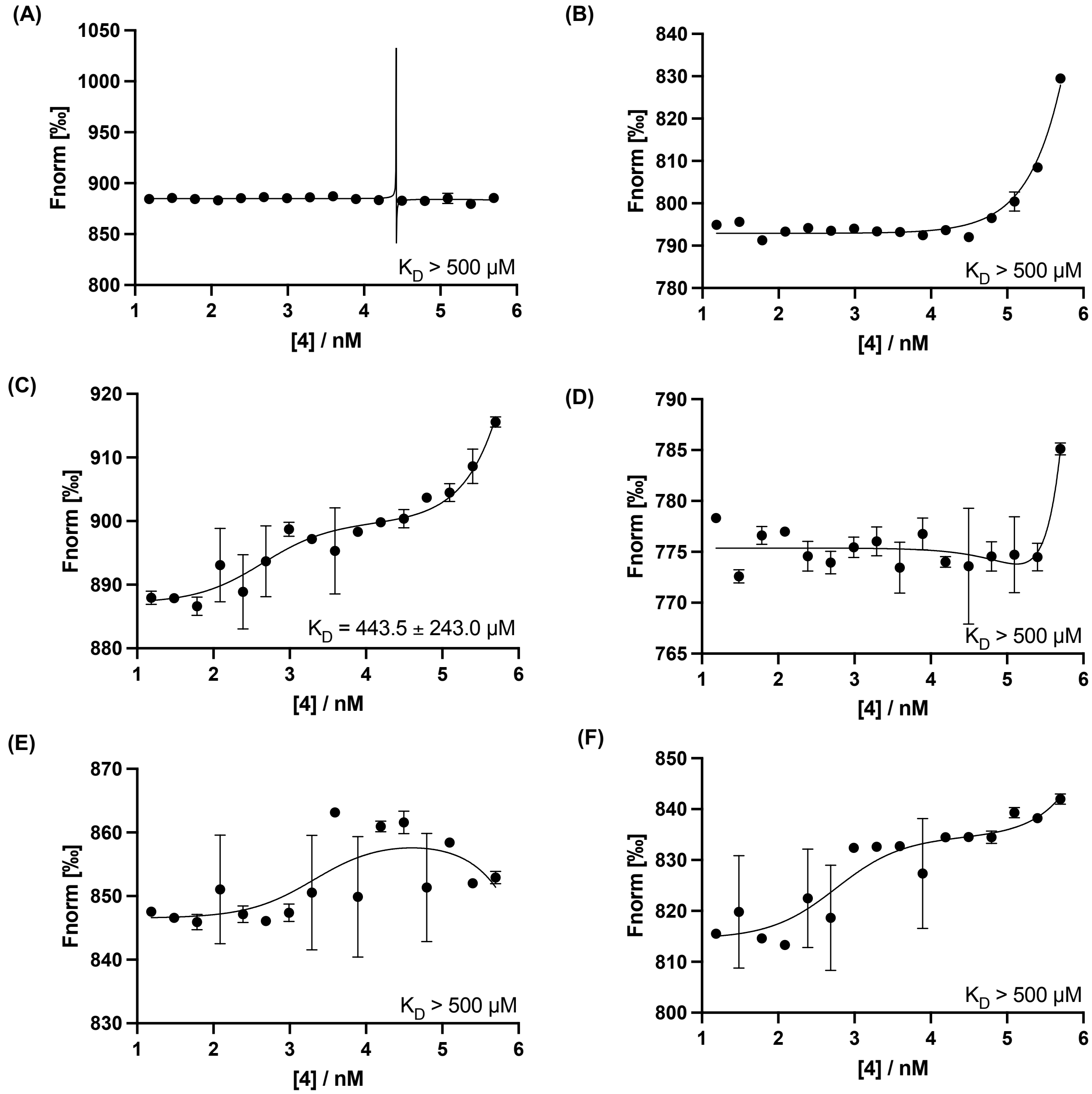

Figure S4. Functional activity of compounds in vitro. Transcription termination assay on *Ssa*-$PreQ_1$ riboswitch in the presence of increasing concentrations of **4** (A) and **8** (B). RT: Full transcript read-through, T: transcription halt due to termination. Shown are the representative PAGE gels from three independent replicates.

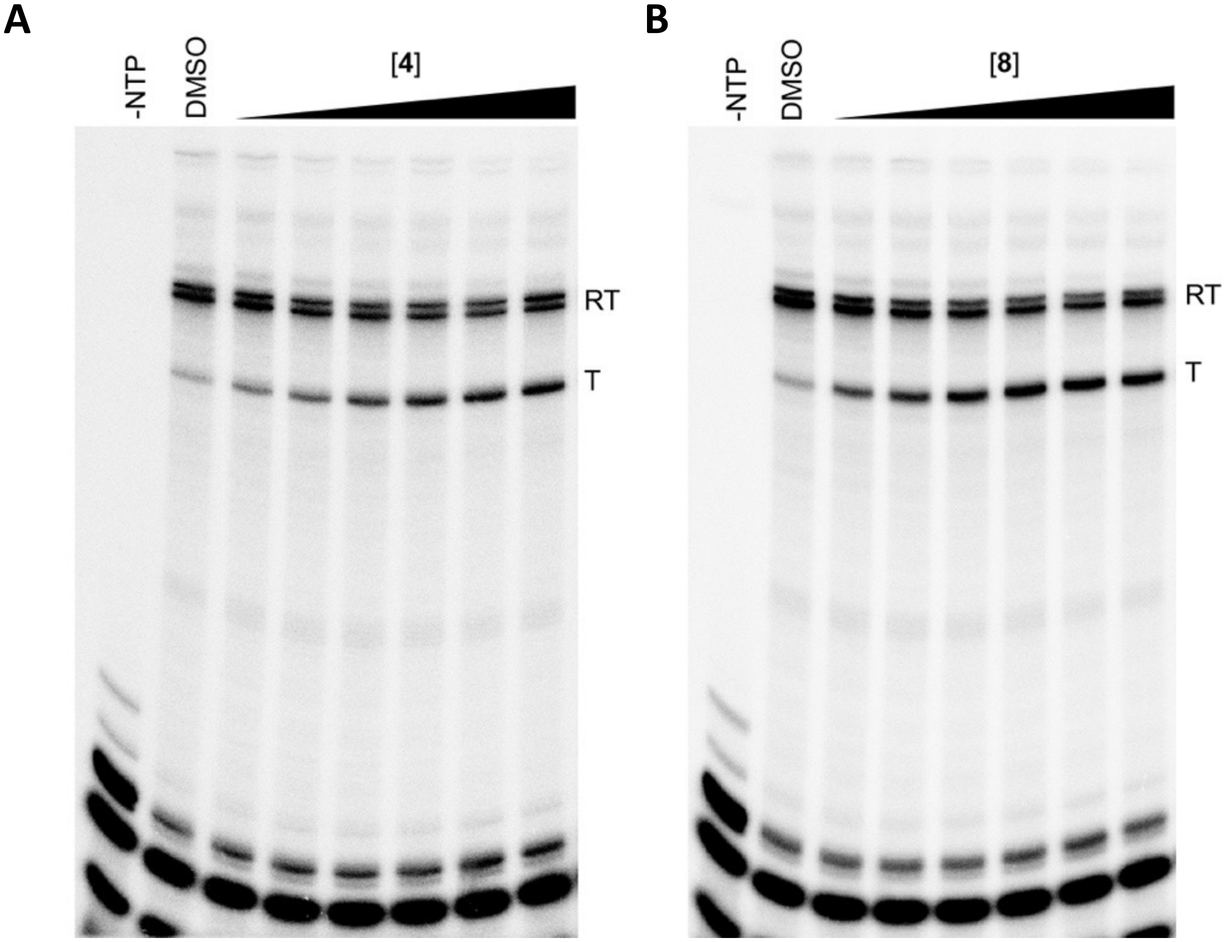

Table S4. **Summary of data collection and refinement statistics.**

	ab13_14_15-**4**	ab13_14_15-**8**
**Data collection**		
Space group	$P6_322$	$P6_322$
Cell dimensions		
a, b, c (Å)	115.9, 115.9, 58.3	116.4, 116.4, 57.8
Wavelength (Å)	1.0	1.0
Resolution (Å)	41.1-2.15 (2.28-2.15)	41.0-2.25 (2.39-2.25)
R_{merge}[a]	0.083 (3.36)	0.064 (3.62)
$R_{p.i.m.}$[b]	0.015 (0.585)	0.012 (0.641)
$CC_{1/2}$[c]	0.999 (0.595)	0.999 (0.589)
$<I>/<\sigma I>$	29.8 (1.57)	32.4 (1.44)
Completeness (%)	99.6 (99.2)	99.9 (99.9)
Redundancy	33.2 (33.3)	31.2 (31.9)
**Refinement**		
Resolution (Å)	41.1-2.15	41.0-2.25
No. reflections	12,983	11,405
R_{work}[d]/R_{free}[e]	0.184/0.193	0.198/0.214
No. atoms		
RNA	667	663
Ligand	21	20
Water	13	5
B-factors (Å^2)		
RNA	77.5	97.6
Ligand	68.6	75.0
Water	70.2	87.4
R.m.s. deviations		
Bond lengths (Å)	0.005	0.002
Bond angles (º)	0.982	0.372
PDB ID	8YAM	8YAN

The values in parentheses are for the outermost shell.

[a] $R_{merge} = \Sigma_{hkl} \Sigma_i |I_i(hkl) - <I(hkl)>| / \Sigma_{hkl} \Sigma_i I_i(hkl)$, where $I_i(hkl)$ is the observed intensity and $\Sigma I I(\text{hkl})>$ is the average intensity over symmetry-equivalent measurements.

[b] Definition of $R_{p.i.m.}$[1]

[c] Pearson correlation coefficient between intensities of random half-dataset. [2]

[d] $R_{work} = \Sigma|F_o - F_c|/\Sigma F_o$ for reflections of working set.

[e] $R_{free} = \Sigma|F_o - F_c|/\Sigma F_o$ for reflections of test set (5.0% of total reflections).

Figure S5. Differences in SHAPE-MaP reactivities. Delta (Δ) SHAPE profiles of (A) *Bsu*-PreQ$_1$ in presence of PreQ$_1$ ligand, *Spn*-PreQ$_1$ with PreQ$_1$ (B) and **4** (C), *Lrh*-PreQ$_1$ with PreQ$_1$ (D) and **4** (E), *Fpn*-PreQ$_1$ with PreQ$_1$ (F) and **4** (G) and *Tte*-PreQ$_1$ in the presence of PreQ$_1$ ligand (H). Shown here are the delta SHAPE profiles of riboswitches with significant changes only. Green bars represent overall stabilization and purple bars show destabilization in the region in the presence of ligand. The nucleotides corresponding to these regions are numbered over the bars. The secondary structure can be found at the left of the delta SHAPE output. Shown are representative images from three technical replicates.

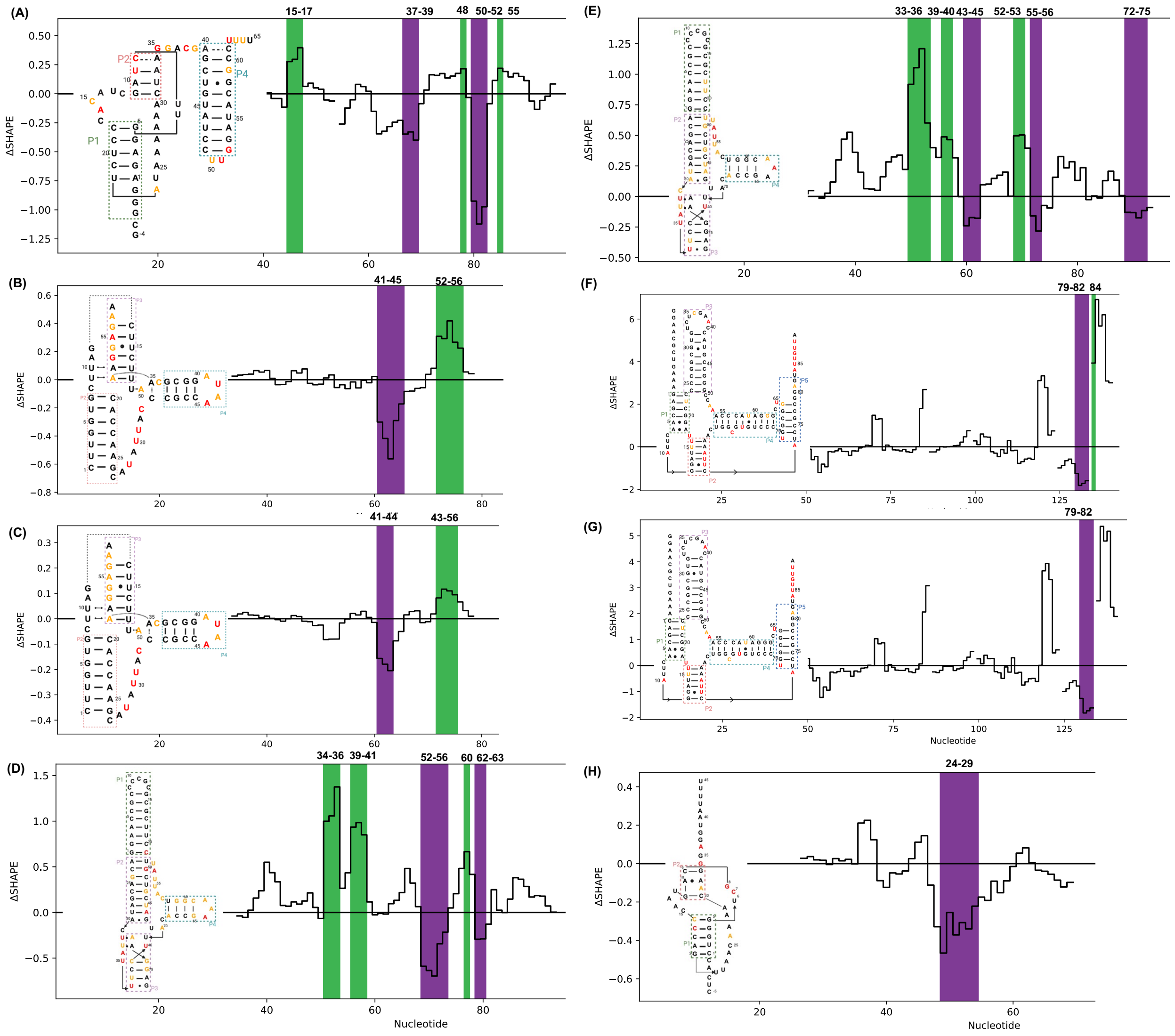

Figure S6. (A) Scheme showing the different MFS experimental modes used. In ramp experiments, the molecules are subjected to cycles of gradually increased and decreased force, whilst in stepped constant force experiments the force is held constant for a fixed period before being increased stepwise. In constant force experiments, the molecules are subjected to a single constant force for an extended period, allowing the transition between different states to be observed. The effect of the binding of stabilizing and destabilizing ligands is indicated. (B) The normalized force distribution for the refolding force, comparing DMSO (control), 500 μM **4** and 500 nM $PreQ_1$ ligand. (C-D) Dose-response curve of the refolding force with increasing concentration of **4** (C) and $PreQ_1$ (D). (E) Another example of the raw trace of a constant force experiment for a single molecule in the presence of DMSO, 500 μM **4** and 500 nM $PreQ_1$. The force is held at the equilibrium force, and the Z-position is plotted against time. (F) Fitting of the probability density of folded lifetimes of a single molecule in constant force experiments. The probability in log scale was plotted against time, and the overall fitting is shown in orange lines. In the $PreQ_1$ condition, two distributions can be fitted, depicted as the two dotted lines. (G) The normalized unfolding rate (1/ folded lifetime) with increasing concentration of $PreQ_1$ ligand. Both the short- and the long-folded lifetimes are plotted in the same graph. (H-I) The refolding rate (1/unfolded lifetime) of the $PreQ_1$ riboswitch with increasing concentrations of $PreQ_1$ (H) or **4** (I), from constant force experiments.

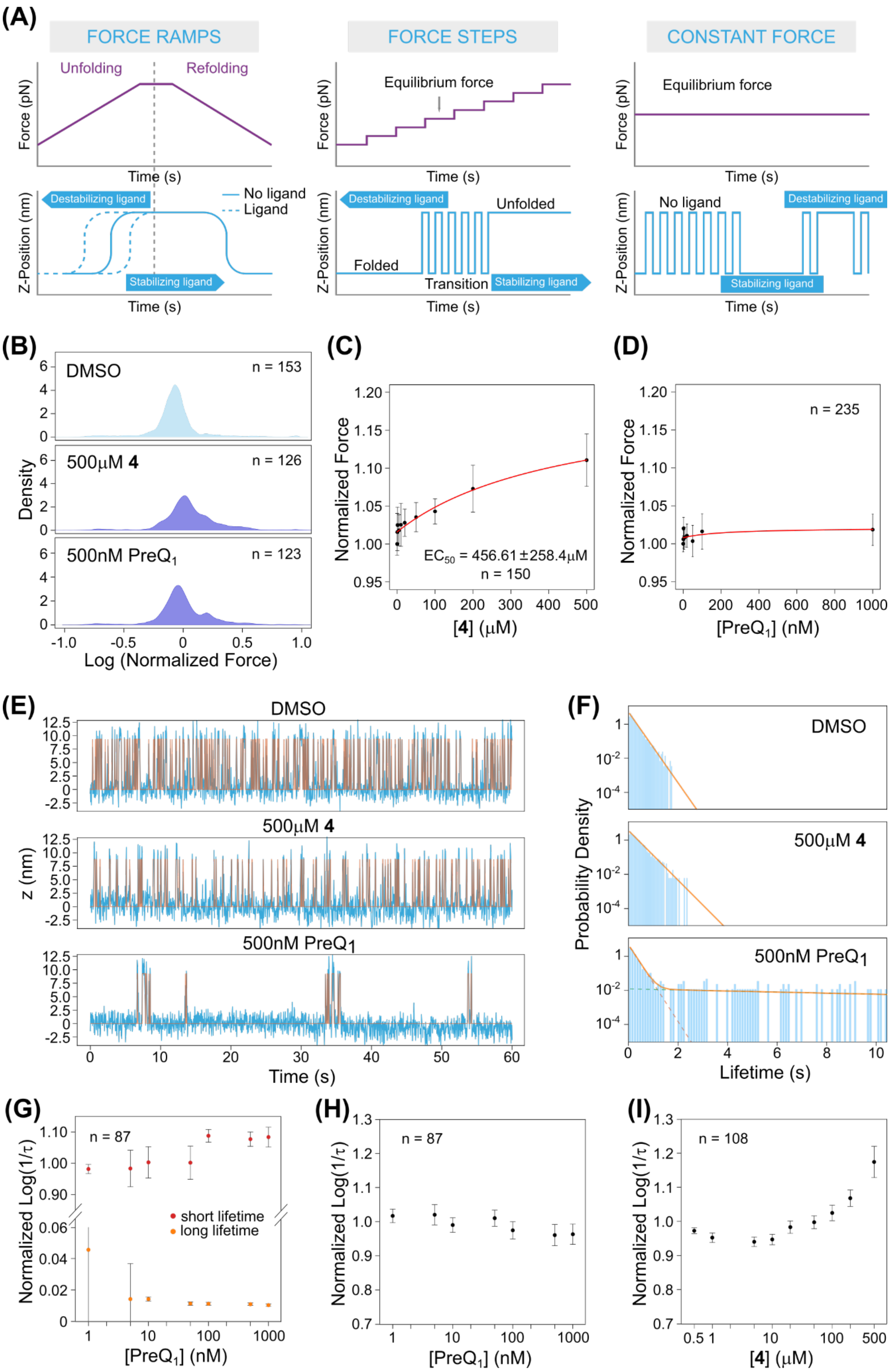

(A)
FORCE RAMPS
FORCE STEPS
CONSTANT FORCE
Unfolding
Refolding
Force (pN)
Time (s)
Equilibrium force
Destabilizing ligand
Stabilizing ligand
No ligand
Ligand
Unfolded
Folded
Transition
Z-Position (nm)
(B)
DMSO
n = 153
500µM 4
n = 126
500nM PreQ1
n = 123
Density
Log (Normalized Force)
(C)
Normalized Force
EC50 = 456.61±258.4µM
n = 150
[4] (µM)
(D)
n = 235
[PreQ1] (nM)
(E)
DMSO
500µM 4
500nM PreQ1
z (nm)
Time (s)
(F)
DMSO
500µM 4
500nM PreQ1
Probability Density
Lifetime (s)
(G)
n = 87
short lifetime
long lifetime
Normalized Log(1/τ)
[PreQ1] (nM)
(H)
n = 87
Normalized Log(1/τ)
[PreQ1] (nM)
(I)
n = 108
Normalized Log(1/τ)
[4] (µM)

Figure S7. (A) Example MSF raw traces (RNA extension vs. time) of a single $PreQ_1$ RNA molecule in the control condition (top), with 500 μM **4** (middle), and 500 nm of $PreQ_1$ ligand (bottom). A higher time scale view of the period framed by the red boxed area is shown for each condition. (B) A different $PreQ_1$ structure, with control (top), and 1 nm i.e., the lowest tested $PreQ_1$ concentration (middle), and 5 nm (bottom) of $PreQ_1$ ligand.

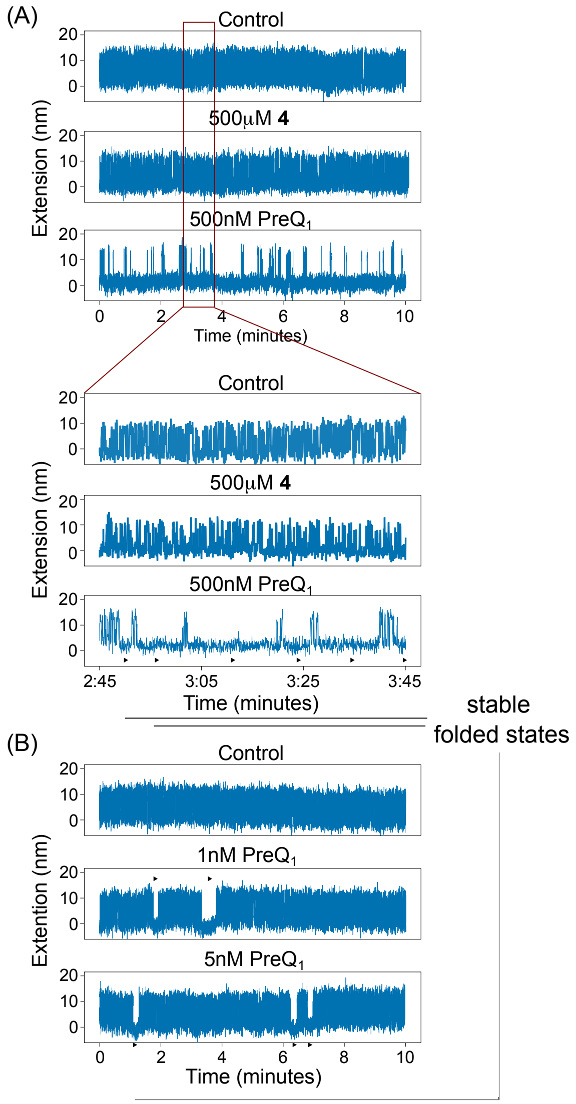

General chemistry methods:

All chemical reagents were obtained from commercial suppliers and used without further purification. Solvents were removed using a Buchi rotary evaporator under reduced pressure. Flash column chromatography was performed using a Teledyne ISCO CombiFlash Rf automated chromatography system.

High resolution mass spectrometry data were acquired on an Agilent 6520 Accurate-Mass Q-TOF LC/MS System, (Agilent Technologies, Inc.) equipped with a dual electro-spray source, operated in the positive-ion mode. Separation was performed on Zorbax 300SB-C18 Poroshell column (2.1 mm x 150 mm; particle size 5 mm). The analytes were eluted using a water/acetonitrile gradient with 0.1% formic acid. Data were acquired at high resolution (1,700 *m/z*), 4 GHz. To maintain mass accuracy during the run time, an internal mass calibration sample was infused continuously during the LC/MS runs. Data acquisition and analysis were performed using MassHunter Workstation Data Software, LCMS Data Acquisition (version B.06.01) and Qualitative Analysis (version B.07.00).

1.

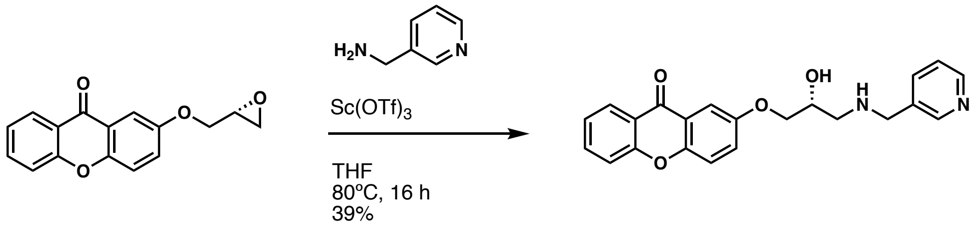

To a solution of 2-(oxiran-2-ylmethoxy)xanthen-9-one (40 mg, 0.15 mmol) in THF (3 mL) was added 3-pyridinemethanamine (0.018 mL) and scandium (III) triflate (4 mg). The mixture was heated to 80ºC and stirred for 16 hours, then was returned to room temperature. The mixture was concentrated down in vacuo, and the crude product was purified by HPLC to afford 22 mg of the desired product (39%). ^{1}H NMR (500 MHz, DMSO-d_6): δ 9.20 (d, J = 30.9 Hz, 2H), 8.72 (d, J = 49.8 Hz, 2H), 8.20 (dd, J = 7.9 Hz, 1H), 8.05 (d, J = 7.8 Hz, 1H), 7.90-7.86 (m, 1H), 7.68 (d, J = 9.1 Hz, 1H), 7.67 (d, J = 8.2 Hz, 1H), 7.60 (d, J = 3.2 Hz, 1H), 7.56 (q, J = 4.8, 3.0 Hz, 1H), 7.51-7.47 (m, 2H), 4.31 (s, 2H), 4.28-4.23 (m, 1H), 4.14-4.09 (m, 2H), 3.23 (s, 1H), 3.08 (s, 1H); ^{13}C NMR (125 MHz, DMSO-d_6): δ 175.8, 155.6, 154.7, 150.6, 150.5, 149.4, 146.8, 139.0, 135.5, 128.2, 126.0, 125.0, 124.3, 121.5, 120.5, 119.9, 118.2, 107.0, 70.4, 64.9, 49.0, 47.8; HRMS: (ESI+) m/z calculated for $C_{22}H_{20}N_2O_4$ [M+H]$^+$: 377.1496, found: 377.1496.

2.

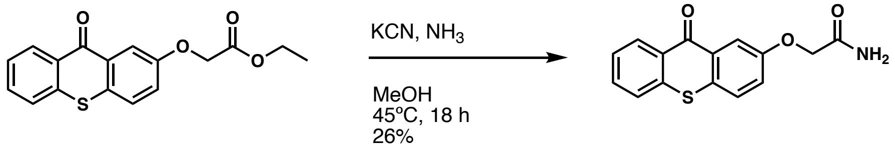

In a sealed microwave vial, a solution of ethyl 2-[(9-oxo-9*H*-thioxanthen-2-yl)oxy]acetate (50 mg, 0.16 mmol), potassium cyanide (2 mg, 0.032 mmol), and ammonia in methanol (2 mL) was heated to 45ºC and stirred for 18 hours. The mixture was returned to room temperature, then the vial and solution were purged with nitrogen. After removing the solvent in vacuo, the resulting crude product was redissolved in dichloromethane and washed with water (10 mL). The organics were extracted with dichloromethane (3 x 10 mL), washed with brine (10 mL), then dried over $MgSO_4$. The crude product was concentrated down onto silica gel, then purified by ISCO Flash column chromatography (DCM:MeOH, 0-40%) to afford 12 mg of the desired product (26%). ^1H NMR (500 MHz, DMSO-d_6): δ 8.48 (dd, *J* = 8.2 Hz, 1H), 7.93 (d, *J* = 2.9 Hz, 1H), 7.86 (dd, *J* = 8.1 Hz, 1H), 7.84 (d, *J* = 8.8 Hz, 1H), 7.79-7.76 (m, 1H), 7.67 (s, 1H), 7.61-7.57 (m, 1H), 7.49 (dd, *J* = 8.8 Hz, 1H), 7.43 (s, 1H), 4.60 (s, 2H); ^{13}C NMR (125 MHz, DMSO-d_6): δ 178.4, 169.4, 156.7, 136.8, 132.8, 129.4, 129.1, 128.8, 128.1, 127.7, 126.7, 126.6, 122.8, 111.7, 66.8; HRMS: (ESI+) m/z calculated for $C_{15}H_{11}NO_3S$ $[M+H]^+$: 286.0532, found: 286.0531.

3.

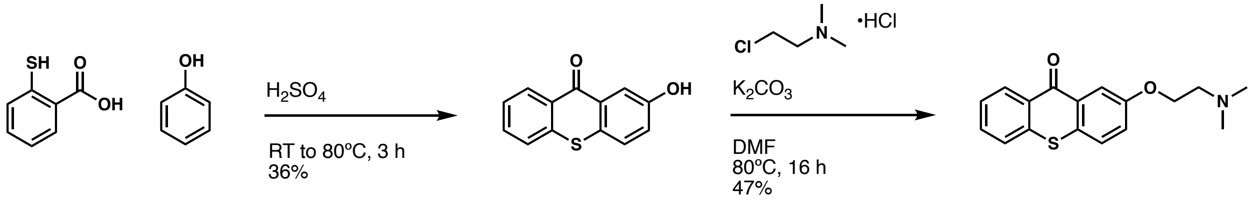

2-Mercaptobenzoic acid (4 g, 25.9 mmol) was slowly added to sulfuric acid (40 mL) at room temperature in a round bottom flask equipped with a magnetic stir bar. The mixture stirred for 15 minutes, then phenol (9.8 mL, 111.5 mmol) was slowly added over 15 minutes. The reaction mixture was then heated to 80ºC and continued stirring for 3 hours. The reaction was diluted with 700 mL of boiling water then allowed to cool to room temperature. The resulting precipitate was then collected by filtration to obtain 2.14 g of 2-hydroxythioxanthone (36%). ^1H NMR (500 MHz,

DMSO-d_6): δ 8.46 (dd, J = 7.9 Hz, 1H), 7.86 (d, J = 2.8 Hz, 1H), 7.82 (dd, J = 8.0 Hz, 1H), 7.77-7.73 (m, 1H), 7.70 (d, J = 8.7 Hz, 1H), 7.58-7.54 (m, 1H), 7.28 (dd, J = 8.0 Hz, 1H); ^{13}C NMR (125 MHz, DMSO-d_6): δ 178.6, 137.0, 132.6, 129.6, 129.1, 128.1, 128.0, 127.8, 126.5, 126.4, 126.0, 122.8, 113.2; HRMS: (ESI+) m/z calculated for $C_{13}H_8O_2S$ $[M+H]^+$: 229.0318, found: 229.0319.

To a solution of 2-hydroxythioxanthone (30 mg, 0.131 mmol) in DMF (3 mL) was added 2-chloro-N,N-dimethyl-ethanamine hydrochloride (19 mg, 0.131 mmol) and potassium carbonate (40 mg, 0.288 mmol). The mixture was heated to 80ºC and stirred for 16 hours. After cooling to room temperature, the reaction was diluted with water (10 mL), and the mixture was extracted with dichloromethane (3 x 10 mL). The combined organic layer was then washed with water (10 mL) and brine (10 mL), then dried with $MgSO_4$. The crude product was concentrated down onto silica gel and purified by ISCO Flash Column Chromatography (DCM:MeOH, 5-40%) to afford 18 mg of the desired product (47%). ^{1}H NMR (500 MHz, DMSO-d_6): δ 8.48 (dd, J = 8.2 Hz, 1H), 7.94 (d, J = 2.9 Hz, 1H), 7.85 (dd, J = 8.1 Hz, 1H), 7.81-7.75 (m, 2H), 7.61-7.57 (m, 1H), 7.45 (dd, J = 8.7 Hz, 1H), 4.20 (t, J = 5.7 Hz, 2H), 2.68 (t, J = 5.7 Hz, 2H), 2.24 (s, 6H); ^{13}C NMR (125 MHz, DMSO-d_6): δ 178.5, 157.4, 143.4, 136.8, 132.8, 129.5, 129.1, 128.2, 127.8, 126.6, 126.6, 122.8, 111.1, 66.3, 57.5, 45.6; HRMS: (ESI+) m/z calculated for $C_{17}H_{17}NO_2S$ $[M+H]^+$: 300.1053, found: 300.1058.

4.

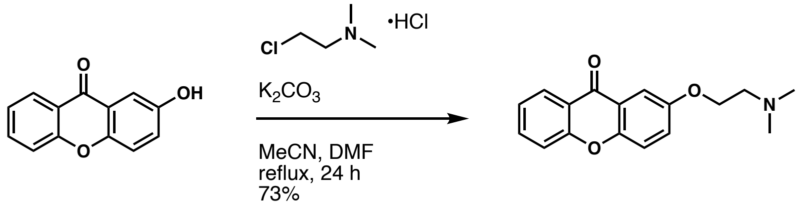

To a solution of 2-hydroxyxanthone (33 mg, 0.156 mmol) in 1:1 acetonitrile and DMF (5 mL) was added 2-chloro-N,N-dimethyl-ethanamine hydrochloride (27 mg, 0.187 mmol) and potassium carbonate (26 mg, 0.187 mmol). The mixture was heated to reflux and stirred for 24 hours. Once cooled to room temperature, the reaction mixture was diluted with water (10 mL), and the mixture was extracted with dichloromethane (3 x 10 mL). The combined organic layer was then washed with water (10 mL) and brine (10 mL), then dried with $MgSO_4$. The crude product was concentrated down onto silica gel and purified by ISCO Flash Column Chromatography (DCM:MeOH, 5-40%) to afford 32 mg of the desired product (73%). ^{1}H NMR (500 MHz, DMSO-

d_6): δ 8.20 (dd, J = 7.9 Hz, 1H), 7.89-7.85 (m, 1H), 7.65 (t, J = 9.1 Hz, 2H), 7.57 (d, J = 3.2 Hz, 1H), 7.50-7.45 (m, 2H), 4.16 (t, J = 5.7 Hz, 2H), 2.67 (t, J = 5.7 Hz, 2H), 2.23 (s, 6H); ^{13}C NMR (125 MHz, DMSO-d_6): δ 175.7, 155.5, 154.9, 150.2, 135.3, 125.9, 124.9, 124.2, 121.5, 120.5, 119.7, 118.1, 106.5, 66.4, 57.5, 45.5; HRMS: (ESI+) m/z calculated for $C_{17}H_{17}NO_3$ $[M+H]^+$: 284.1281, found: 284.1282.

5.

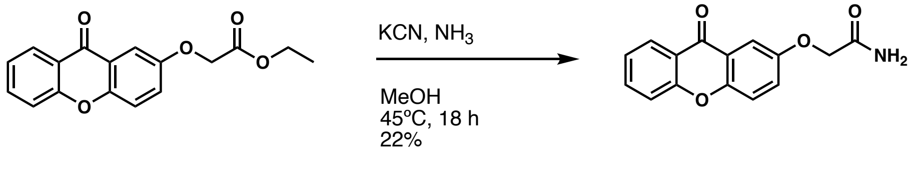

In a sealed microwave vial, a solution of ethyl 2-[(9-oxo-9*H*-xanthen-2-yl)oxy]acetate (50 mg, 0.17 mmol), potassium cyanide (2.2 mg, 0.034 mmol), and ammonia in methanol (2 mL) was heated to 45ºC and stirred for 18 hours. The mixture was returned to room temperature, then the vial and solution were purged with nitrogen. After removing the solvent in vacuo, the resulting crude product was redissolved in dichloromethane and washed with water (10 mL). The organics were extracted with dichloromethane (3 x 10 mL), washed with brine (10 mL), then dried over $MgSO_4$. The crude product was concentrated down onto silica gel, then purified by ISCO Flash column chromatography (DCM:MeOH, 0-40%) to afford 10 mg of the desired product (22%). ^{1}H NMR (500 MHz, DMSO-d_6): δ 8.21 (dd, J = 8.1 Hz, 1H), 7.90-7.87 (m, 1H), 7.70-7.66 (m, 3H), 7.58-7.54 (m, 2H), 7.50-7.47 (m, 1H), 7.42 (s, 1H), 4.58 (s, 2H); ^{13}C NMR (125 MHz, DMSO-d_6): δ 175.8, 169.5, 155.6, 154.3, 150.5, 135.5, 126.0, 125.0, 124.3, 121.4, 120.5, 119.8, 118.2, 107.3, 67.1; HRMS: (ESI+) m/z calculated for $C_{15}H_{11}NO_4$ $[M+H]^+$: 270.0761, found: 270.0757.

6.

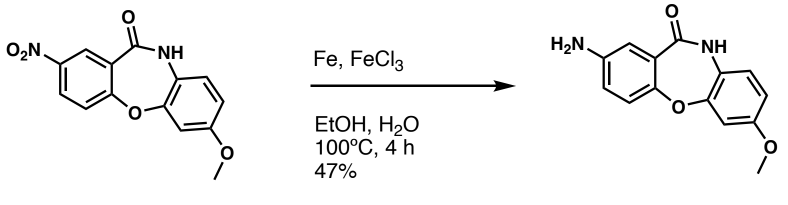

In a round bottom flask, a solution of 7-methoxy-2-nitrodibenz[*b*,*f*][1,4]oxazepin-11(10*H*)-one (150 mg, 0.52 mmol), iron (24 mg, 0.42 mmol), and iron (III) chloride (4.2 mg, 0.03 mmol) in ethanol (2

mL) and water (2 mL) was heated to 100ºC while stirring. After 2.5 hours, additional iron powder (24 mg, 0.42 mmol) was added, and the mixture continued stirring for 1.5 hours. The mixture was returned to room temperature and filtered through a pad of celite. The crude product was purified by ISCO Flash column chromatography (DCM:MeOH, 0-40%) to afford 62 mg of the desired product (47%). ^{1}H NMR (500 MHz, DMSO-d_6): δ 10.10 (s, 1H), 7.02 (d, *J* = 8.6 Hz, 1H), 6.98 (d, *J* = 8.5 Hz, 1H), 6.91 (d, *J* = 2.7 Hz, 1H), 6.84 (d, *J* = 2.7 Hz, 1H), 6.73-6.70 (m, 2H), 5.18 (s, 2H), 3.72 (s, 3H); ^{13}C NMR (125 MHz, DMSO-d_6): δ 166.1, 156.7, 152.3, 149.3, 146.2, 125.9, 124.4, 122.1, 120.8, 118.8, 114.6, 111.0, 106.3, 55.5; HRMS: (ESI+) m/z calculated for $C_{14}H_{12}N_2O_3$ $[M+H]^+$: 257.0921, found: 257.0925.

7.

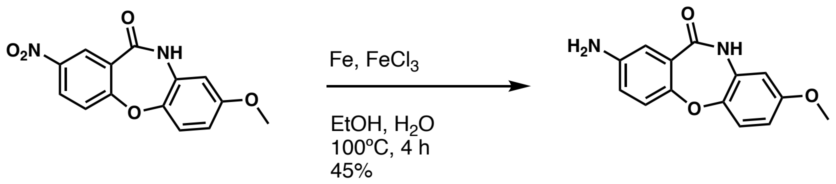

In a round bottom flask, a solution of 8-methoxy-2-nitrodibenz[*b*,*f*][1,4]oxazepin-11(10*H*)-one (150 mg, 0.52 mmol), iron (24 mg, 0.42 mmol), and iron (III) chloride (4.2 mg, 0.03 mmol) in ethanol (2 mL) and water (2 mL) was heated to 100ºC while stirring. After 2.5 hours, additional iron powder (24 mg, 0.42 mmol) was added, and the mixture continued stirring for 1.5 hours. The mixture was returned to room temperature and filtered through a pad of celite. The crude product was purified by ISCO Flash column chromatography (DCM:MeOH, 0-40%) to afford 60 mg of the desired product (45%). ^{1}H NMR (500 MHz, DMSO-d_6): δ 10.21 (s, 1H), 7.13 (d, *J* = 8.8 Hz, 1H), 6.95 (d, *J* = 8.5 Hz, 1H), 6.91 (d, *J* = 2.9 Hz, 1H), 6.72 (dd, *J* = 8.8 Hz, 1H), 6.67 (d, *J* = 2.9 Hz, 1H), 6.64 (dd, *J* = 8.8 Hz, 1H), 5.16 (s, 2H), 3.69 (s, 3H); ^{13}C NMR (125 MHz, DMSO-d_6): δ 166.5, 156.3, 149.6, 146.0, 145.0, 132.0, 125.7, 121.3, 120.6, 119.0, 114.6, 109.6, 106.6, 55.5; HRMS: (ESI+) m/z calculated for $C_{14}H_{12}N_2O_3$ $[M+H]^+$: 257.0921, found: 257.0919.

8.

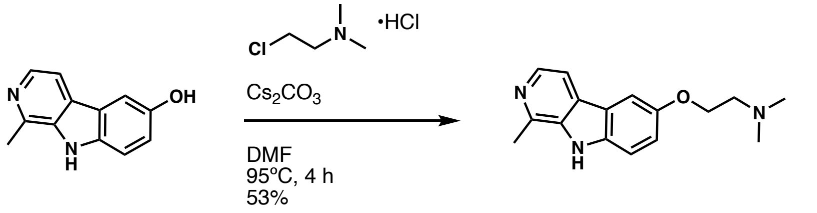

To a solution of harmol (25 mg, 0.126 mmol) in DMF (2.5 mL) was added 2-chloro-N,N-dimethyl-ethanamine hydrochloride (27 mg, 0.189 mmol) and cesium carbonate (62 mg, 0.189 mmol). The mixture was heated to reflux and stirred for four hours. After cooling to room temperature, the reaction was diluted with water (10 mL), and the mixture was extracted with dichloromethane (3 x 10 mL). The combined organic layer was then washed with brine (10 mL) and dried with Na_2SO_4. The crude product was concentrated down onto silica gel and purified by ISCO Flash Column Chromatography (DCM:MeOH, 5-40%) to afford 18 mg of yellowish brown solid (53%). ^{1}H NMR (500 MHz, DMSO-d_6): δ 11.80 (s, 1H), 8.15 (d, *J* = 5.1 Hz, 1H), 8.07 (d, *J* = 9.0 Hz, 1H), 7.83 (d, *J* = 4.8 Hz, 1H), 7.08 (s, 1H), 6.87 (d, *J* = 9.0 Hz, 1H), 4.34 (t, *J* = 5.0 Hz, 2H), 3.16 (t, *J* = 5.6 Hz, 2H), 2.74 (s, 3H), 2.57 (s, 6H); ^{13}C NMR (125 MHz, DMSO-d_6): δ 158.7, 142.0, 141.3, 137.3, 134.6, 127.3, 122.7, 115.1, 112.0, 109.4, 95.7, 64.3, 56.3, 44.0, 20.3; HRMS: (ESI+) m/z calculated for $C_{16}H_{19}N_3O$ $[M+H]^+$: 270.1601, found: 270.1601.

9.

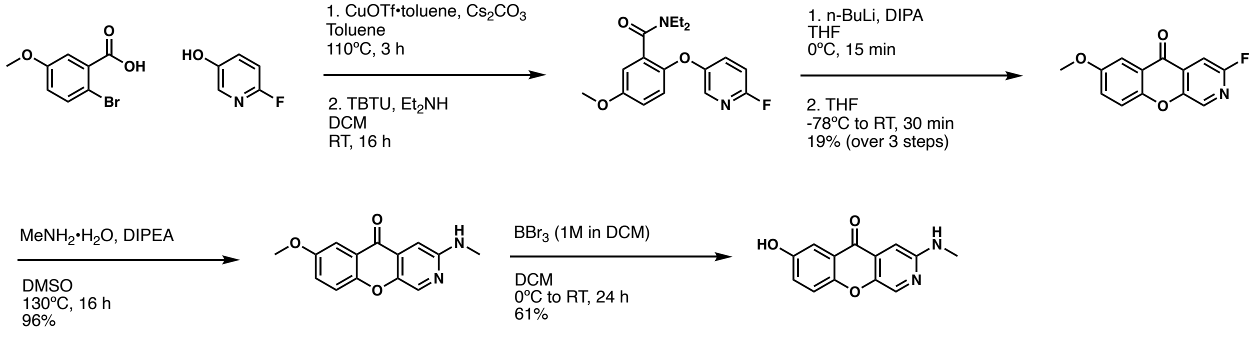

A solution of 2-bromo-5-methoxybenzoic acid (1.5 g, 6.52 mmol), 6-fluoropyridin-3-ol (884 mg, 7.82 mmol), cesium carbonate (4.25 g, 13.04 mmol), and copper (I) trifluoromethanesulfonate toluene complex (103 mg, 0.20 mmol) in toluene (20 mL) was heated to 110ºC and stirred for three hours. The mixture was returned to room temperature, then the liquid layer was decanted. Water (20 mL) and ethyl acetate (10 mL) were added to dissolve the remaining residue. The aqueous phase was neutralized with 6N HCl, then the organic layer was extracted using ethyl

acetate (3 x 10 mL). The organic layer and ethyl acetate washings were washed with brine (15 mL) then dried (Na_2SO_4). The crude product was taken to the next step without further purification. 2-((6-fluoropyridin-3-yl)oxy)-5-methoxybenzoic acid (1.72 g, 6.52 mmol) was dissolved in dichloromethane (30 mL), then TBTU (2.1 g, 6.52 mmol) was added. The stirring solution was slowly treated with diethylamine (1.35 mL, 13.02 mmol), and the mixture continued stirring at room temperature for 16 hours. The reaction was quenched using saturated ammonium chloride (10 mL) and water (15 mL). The organic layer was extracted using dichloromethane (3 x 15 mL), then the organic layer and DCM washings were washed with brine (15 mL) and dried (Na_2SO_4). The crude product was concentrated down in vacuo and taken to the next step.

To a solution of n-butyllithium (2.5 M in hexane) (4.84 mL, 12.1 mmol) in tetrahydrofuran (5 mL) at 0ºC was added diisopropanolamine (1.7 mL, 12.1 mmol), and the mixture stirred for 15 minutes. The resulting mixture was then added to a -78ºC solution of *N*,*N*-diethyl-2-((6-fluoropyridin-3-yl)oxy)-5-methoxybenzamide (700 mg, 2.2 mmol) in tetrahydrofuran (9 mL). The reaction stirred at -78ºC for 30 minutes, then was allowed to warm to room temperature. The reaction was slowly quenched with saturated ammonium chloride (10 mL), then the organic layer was extracted with ethyl acetate (3 x 10 mL). The organic layer and ethyl acetate washings were washed with brine (10 mL), then dried (Na_2SO_4). The crude product was purified by ISCO flash column chromatography (0-100% EtOAc in hexanes) to afford 300 mg of yellow solid (19% over three steps). ^{1}H NMR (500 MHz, DMSO-d_6): δ 8.86 (s, 1H), 7.73 (d, *J* = 3.1 Hz, 1H), 7.71 (d, *J* = 9.1 Hz, 1H), 7.58 (dd, *J* = 9.1, 3.1 Hz, 1H), 7.52 (d, *J* = 3.1 Hz, 1H), 3.89 (s, 3H); ^{13}C NMR (125 MHz, DMSO-d_6): δ 174.9, 159.8-157.5 (d, $J^1_{C\text{-}F}$ = 233.0 Hz), 156.2, 150.3, 149.0 (d, $J^4_{C\text{-}F}$ = 4.4 Hz), 140.4-140.3 (d, $J^3_{C\text{-}F}$ = 16.6 Hz), 129.7-129.6 (d, $J^3_{C\text{-}F}$ = 7.3 Hz), 126.1, 121.0, 120.2, 105.5, 103.0-102.6 (d, $J^2_{C\text{-}F}$ = 40.1 Hz), 55.9; HRMS: (ESI+) m/z calculated for $C_{13}H_8FNO_3$ $[M+H]^+$: 246.0561, found: 246.0561.

A mixture of 3-fluoro-7-methoxy-5*H*-chromeno[2,3-*c*]pyridin-5-one (24 mg, 0.098 mmol), methylamine (40% in H_2O) (0.4 mL), and diisopropylethylamine (35 µL, 0.196 mmol) in dimethyl sulfoxide (1 mL) was heated to 130ºC and stirred for 16 hours. The mixture was returned to room temperature, then diluted with dichloromethane (5 mL) and water (5 mL). The organic layer was extracted with DCM (3 x 10 mL), washed with brine (10 mL), then dried (Na_2SO_4). The product was concentrated down in vacuo to afford 24 mg of yellow solid (96%). ^{1}H NMR (500 MHz, DMSO-d_6): δ 8.66 (s, 1H), 7.61 (dd, *J* = 8.9, 0.7 Hz, 1H), 7.52 (d, *J* = 2.8 Hz, 1H), 7.51 (dd, *J* = 8.9, 3.2 Hz, 1H), 6.94 (s, 1H), 6.76 (q, *J* = 5.0, 4.8 Hz, 1H), 3.87 (s, 3H), 2.84 (d, *J* = 4.7 Hz, 3H); ^{13}C NMR (125 MHz, DMSO-d_6): δ 176.0, 156.5, 155.4, 150.6, 143.2, 139.7, 127.2, 125.6, 120.7, 120.1,

105.4, 96.8, 55.7, 28.6; HRMS: (ESI+) m/z calculated for $C_{14}H_{12}N_2O_3$ $[M+H]^+$: 257.0921, found: 257.0925.

A solution of 7-methoxy-3-(methylamino)-5*H*-chromeno[2,3-*c*]pyridin-5-one (24 mg, 0.094 mmol) in dichloromethane (1 mL) was cooled to 0ºC, then boron tribromide (1M solution in DCM) (0.94 mL, 0.94 mmol) was slowly added. The mixture was slowly returned to room temperature and stirred for 24 hours. The reaction was diluted with ice-cold water (10 mL), then the organic layer was extracted using dichloromethane (3 x 10 mL). The organic layer and DCM washings were washed with brine (15 mL), then dried (Na_2SO_4). The crude product was purified by reverse phase ISCO flash column chromatography (10-100% acetonitrile + 0.1% TFA in water + 0.1% TFA) to afford 14 mg of yellow solid (61%). ^{1}H NMR (500 MHz, DMSO-*d*$_6$): δ 9.93 (s, 1H), 8.63 (s, 1H), 7.51 (d, *J* = 8.6 Hz, 1H), 7.42 (d, *J* = 2.9 Hz, 1H), 7.34 (dd, *J* = 8.9, 3.2 Hz, 1H), 6.91 (s, 1H), 6.73 (q, *J* = 4.8 Hz, 1H), 2.83 (d, *J* = 5.1 Hz, 3H); ^{13}C NMR (125 MHz, DMSO-*d*$_6$): δ 176.0, 156.4, 153.6, 149.5, 143.3, 139.6, 127.2, 125.4, 121.0, 119.7, 108.2, 96.8, 28.7; HRMS: (ESI+) m/z calculated for $C_{13}H_{10}N_2O_3$ $[M+H]^+$: 243.0764, found: 243.0766.

NMR Spectra

1

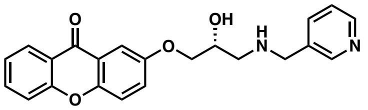

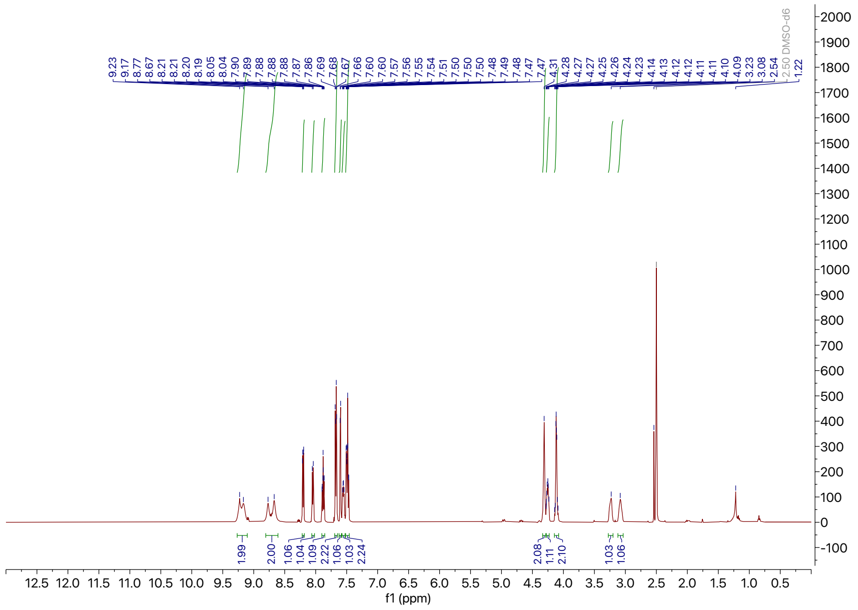

^{1}H NMR. 500 MHz, DMSO-*d*$_6$

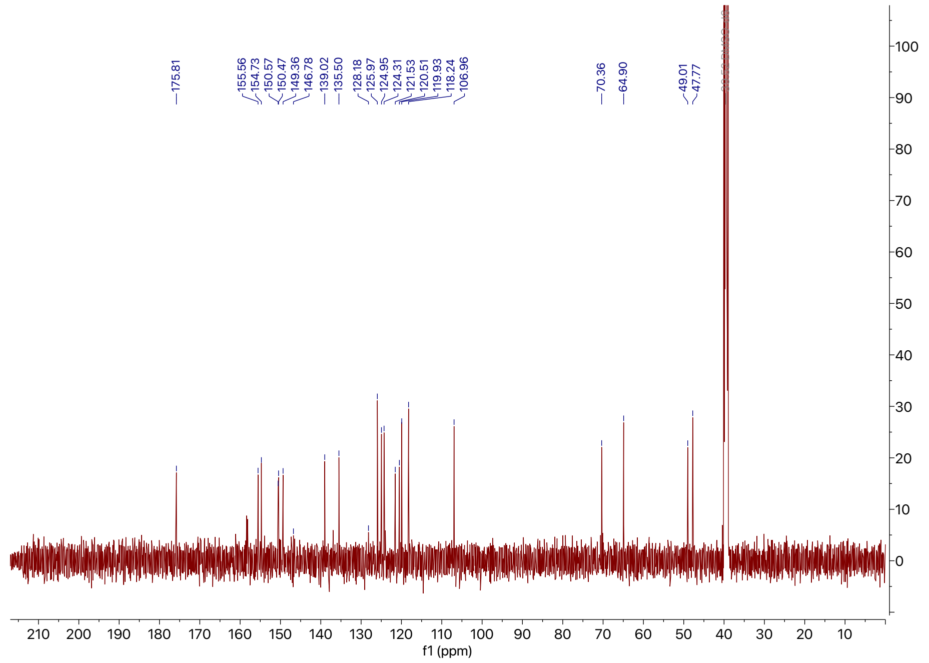

^{13}C NMR. 125 MHz, DMSO-d_6

2

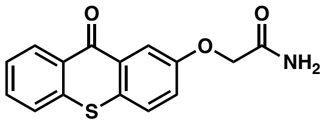

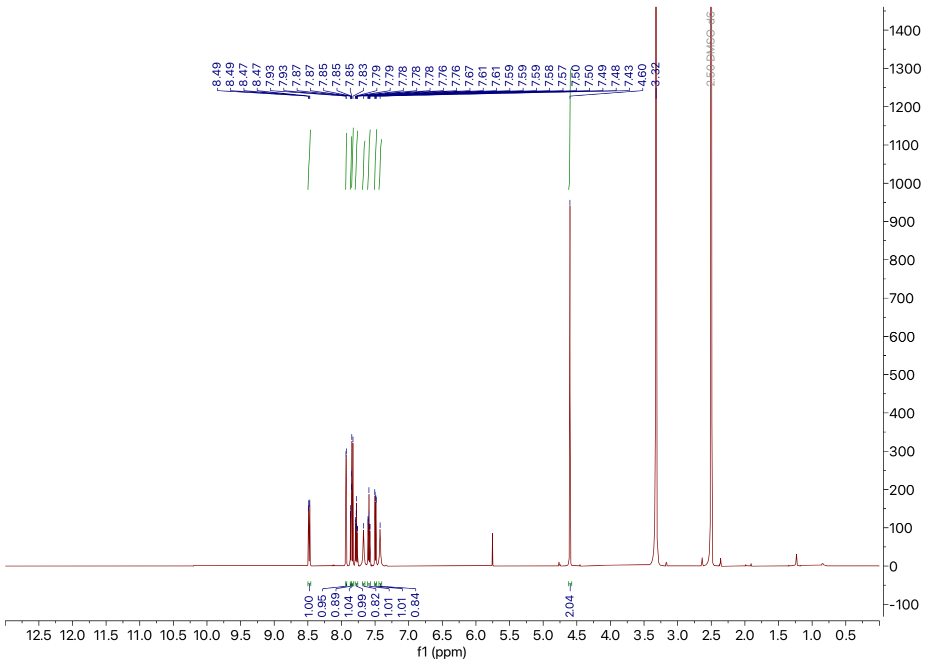

^{1}H NMR. 500 MHz, DMSO-d_6

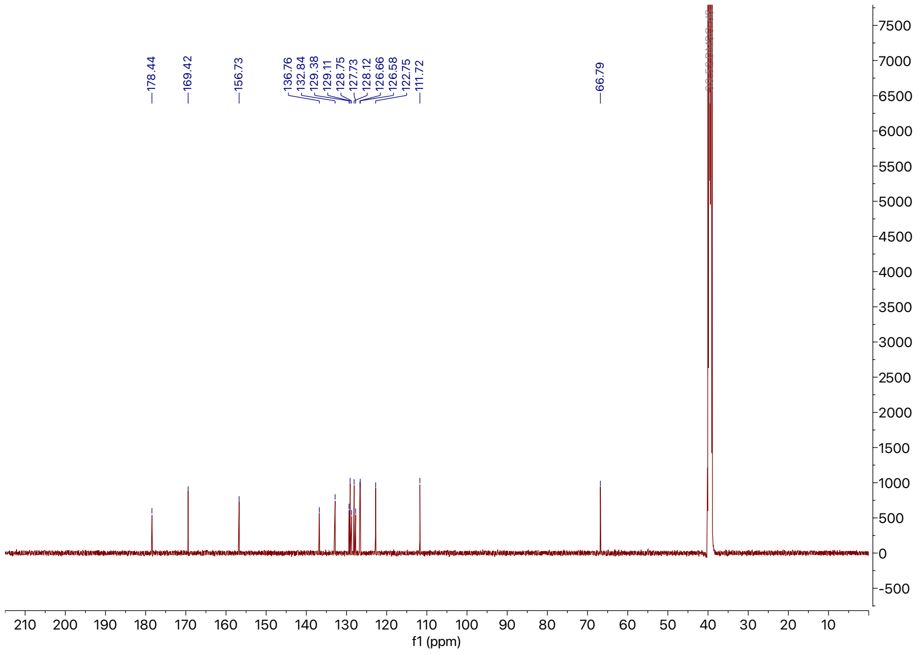

^{13}C NMR. 125 MHz, DMSO-d_6**3-1**

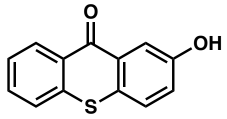

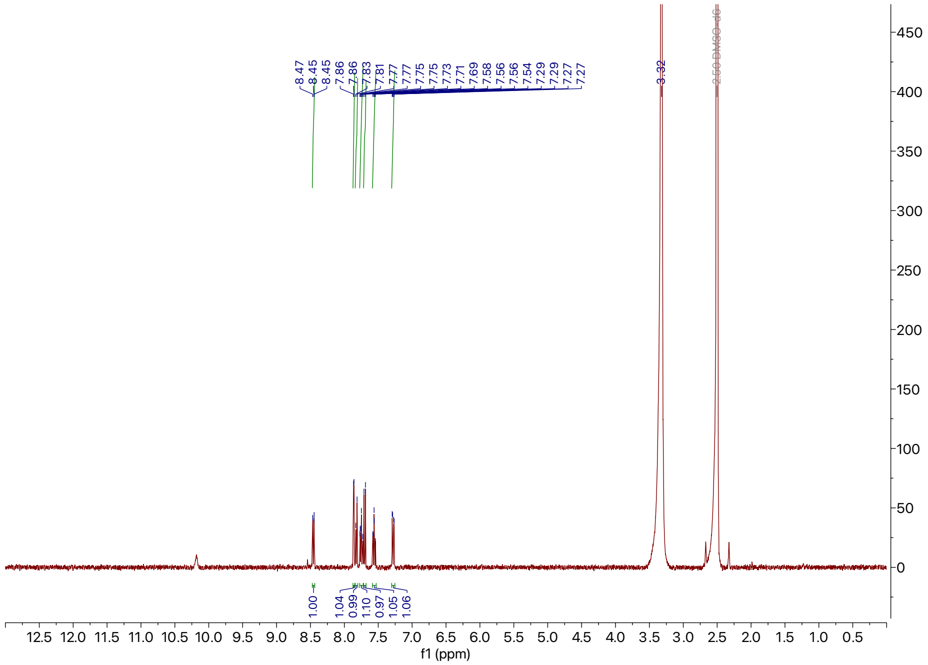

^{1}H NMR. 500 MHz, DMSO-d_6

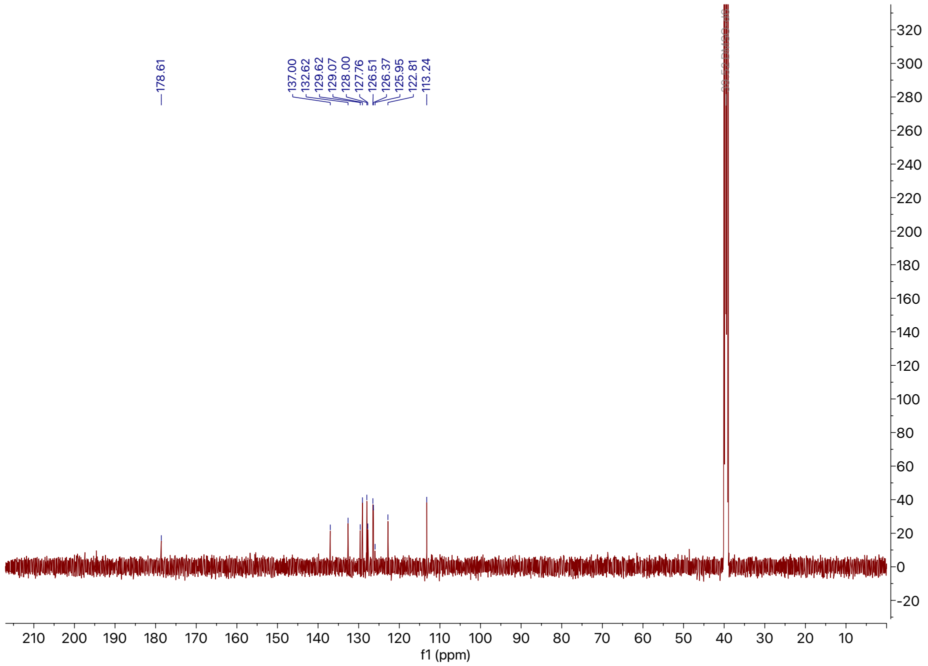

^{13}C NMR. 125 MHz, DMSO-d_6

3-2

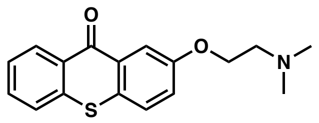

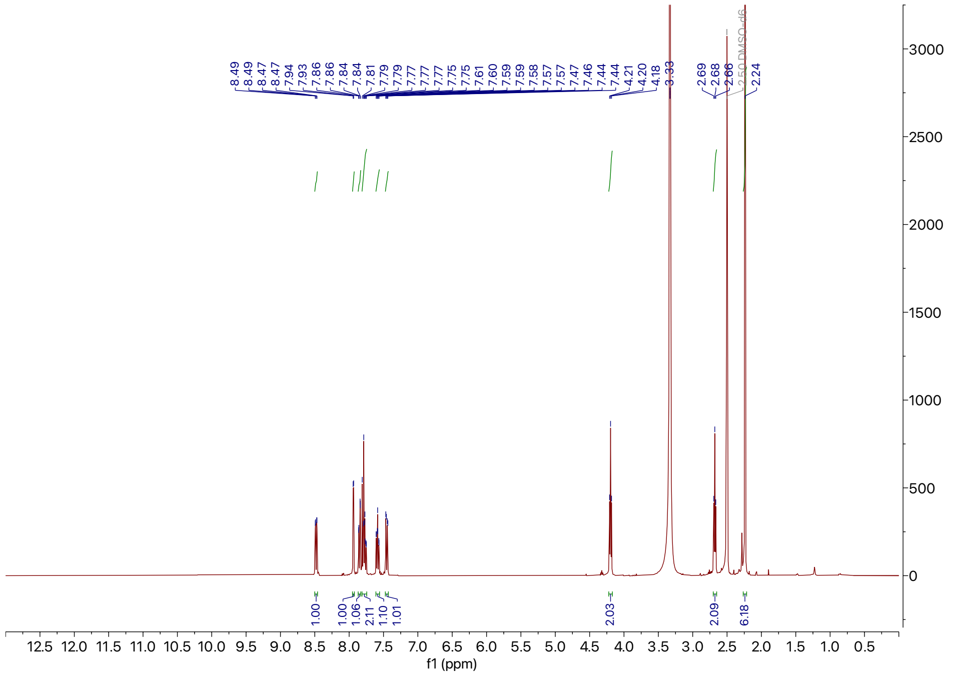

^{1}H NMR. 500 MHz, DMSO-d_6

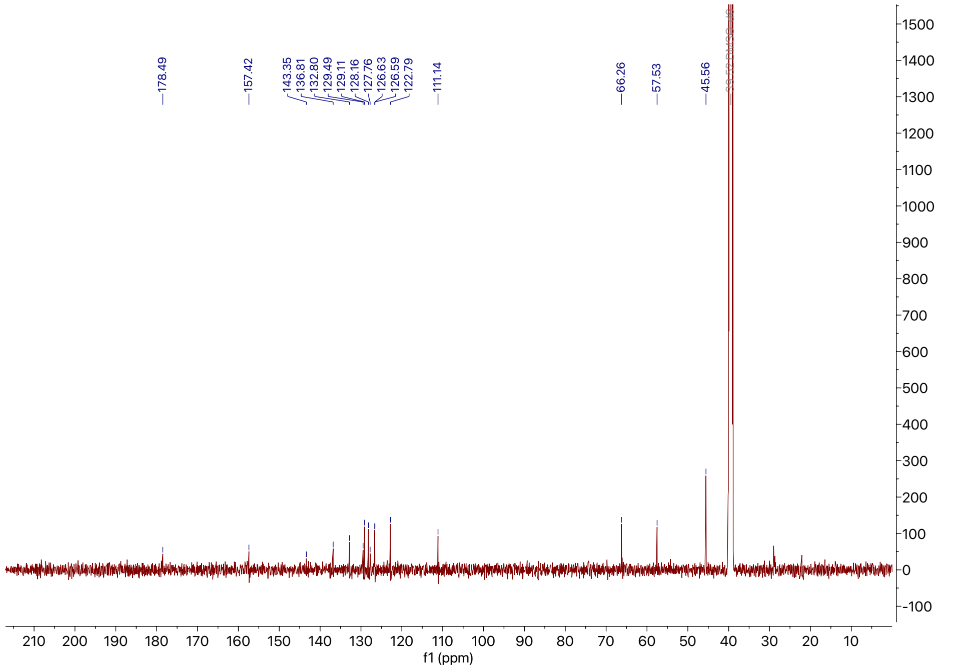

^{13}C NMR. 125 MHz, DMSO-d_6

4

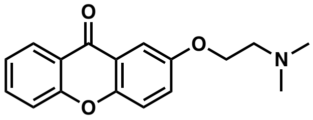

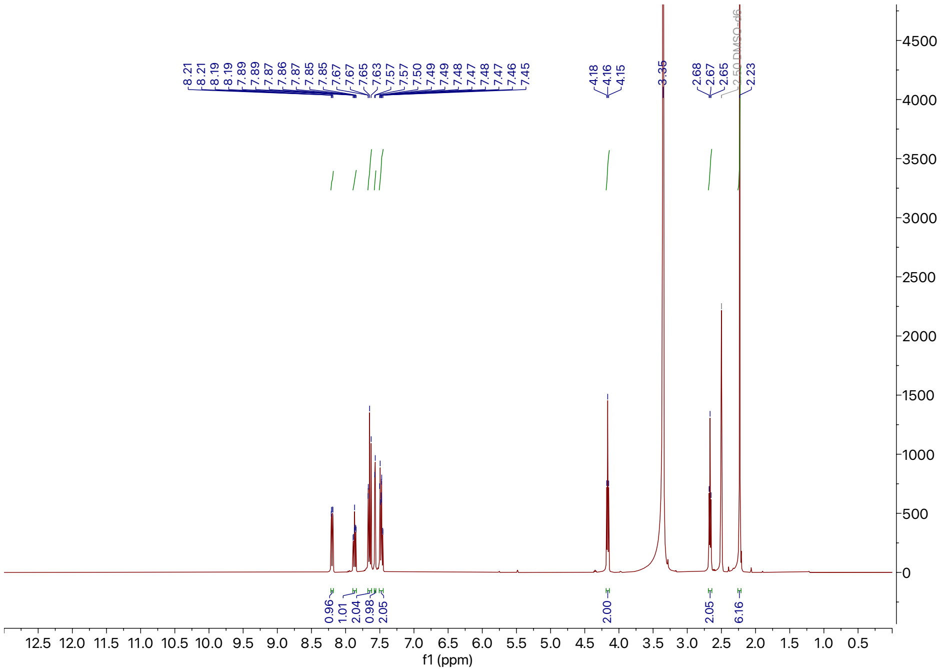

^{1}H NMR. 500 MHz, DMSO-d_6

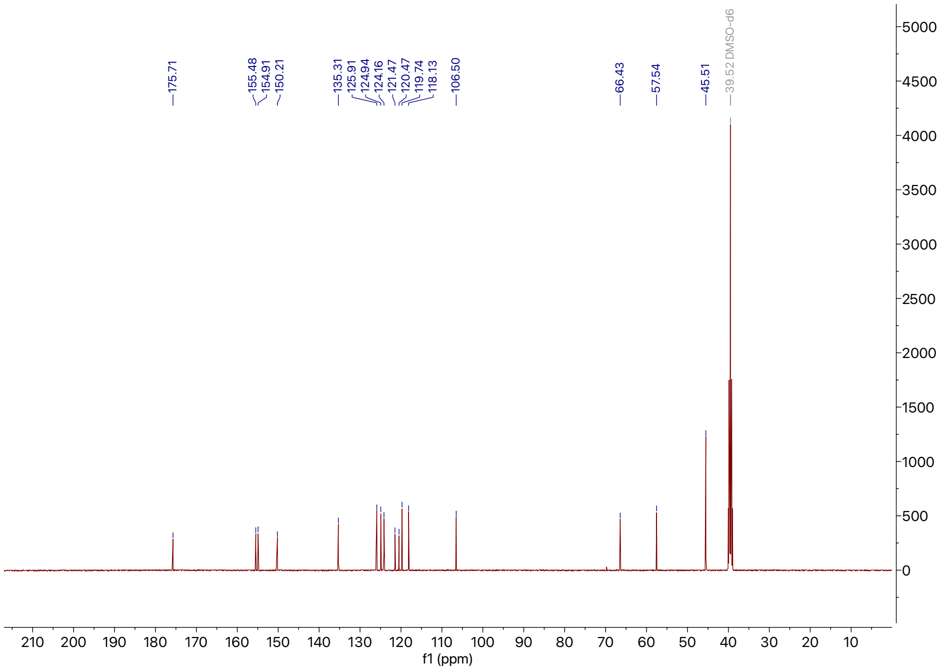

^{13}C NMR. 125 MHz, DMSO-d_6

5

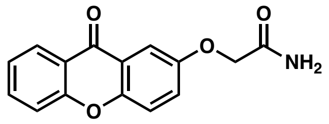

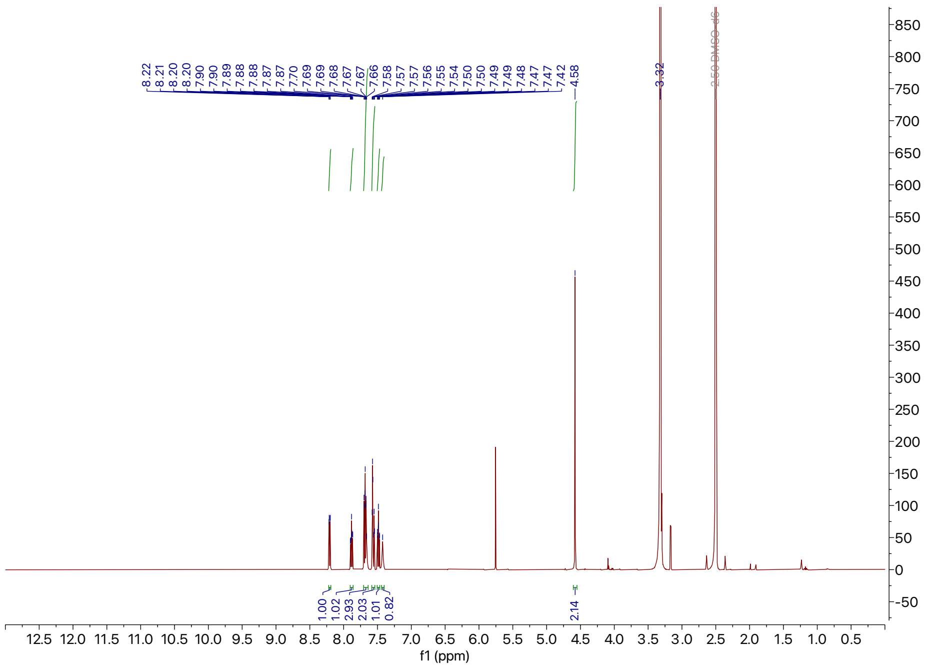

^{1}H NMR. 500 MHz, DMSO-d_6

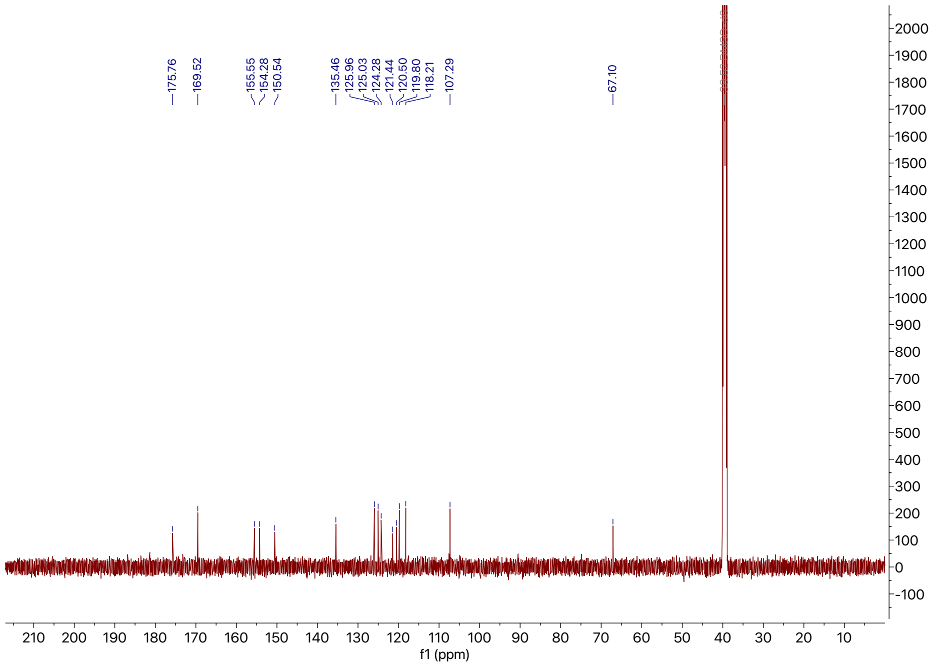

^{13}C NMR. 125 MHz, DMSO-d_6

6

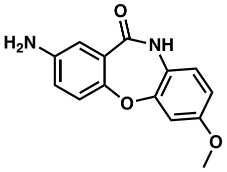

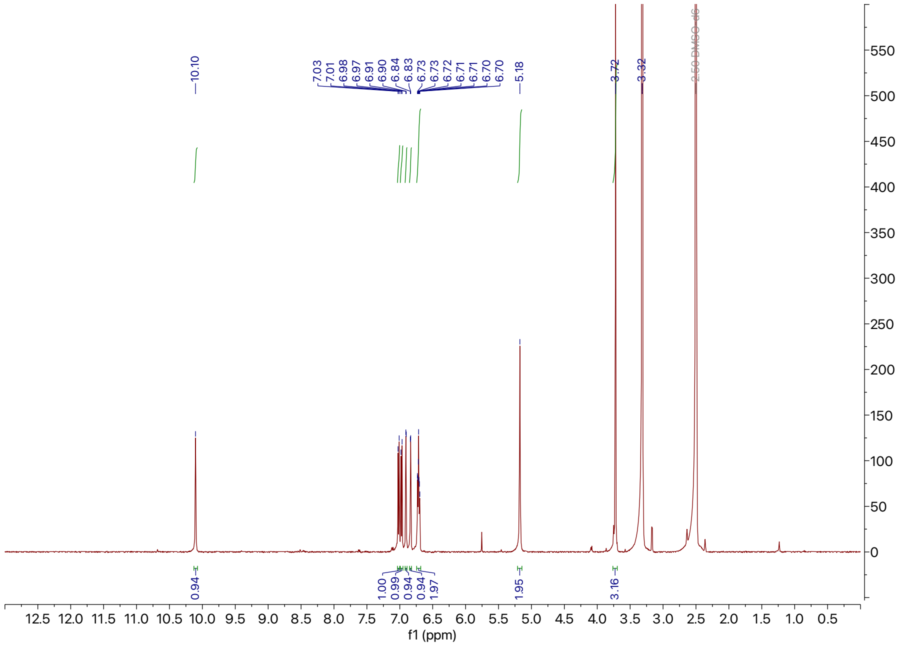

^{1}H NMR. 500 MHz, DMSO-*d*6

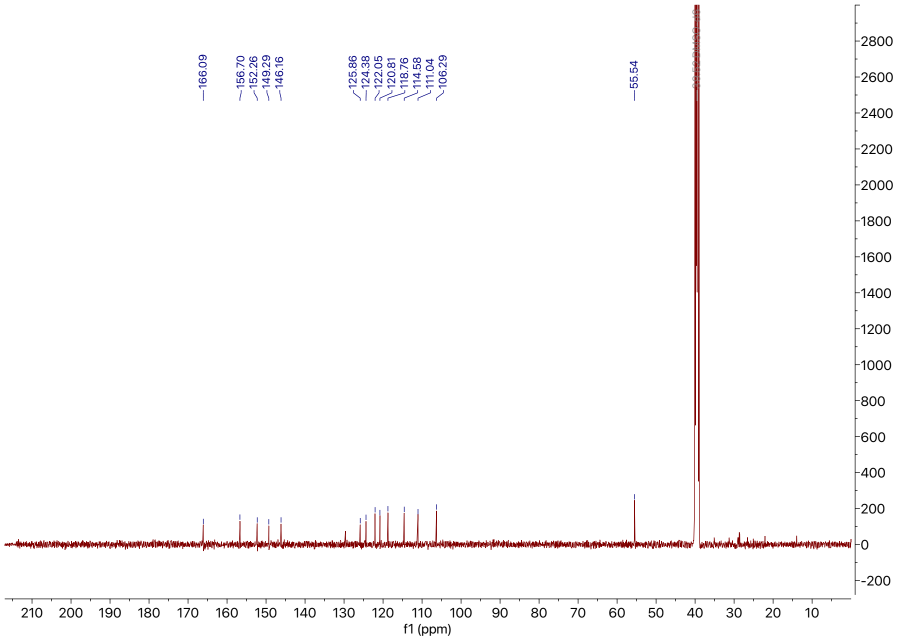

^{13}C NMR. 125 MHz, DMSO-*d*6

7

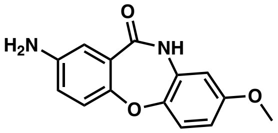

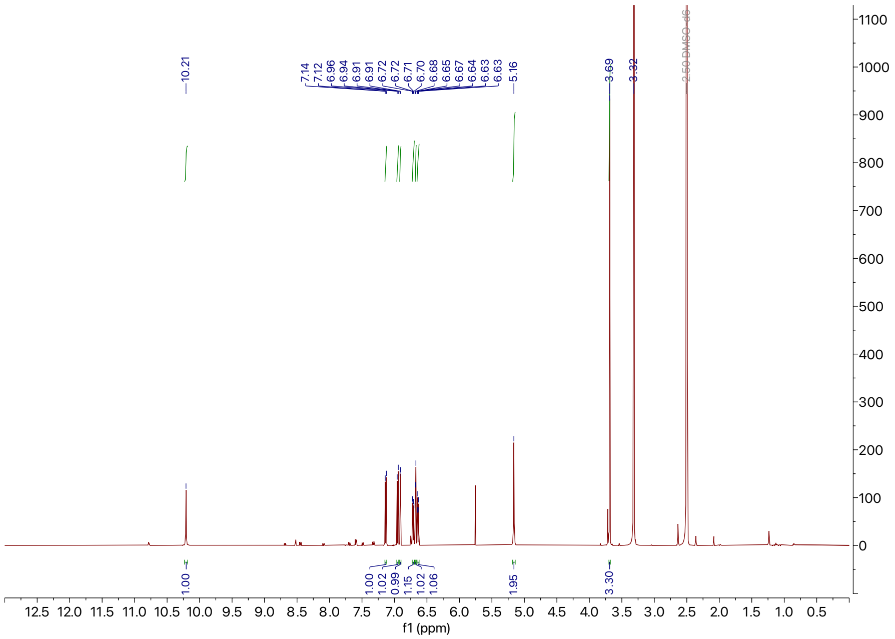

^{1}H NMR. 500 MHz, DMSO-d_6

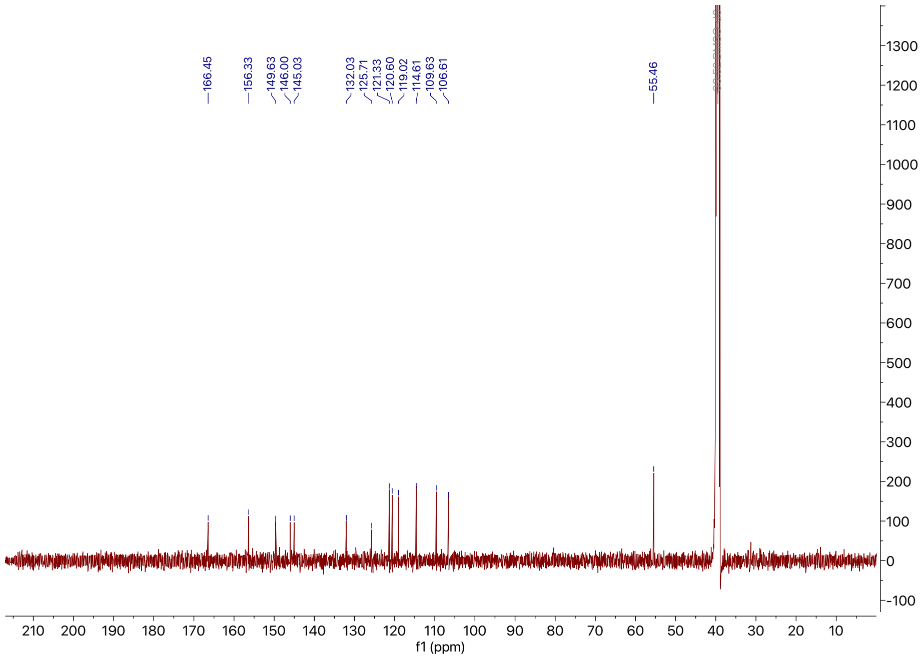

^{13}C NMR. 125 MHz, DMSO-d_6

8

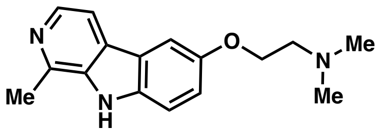

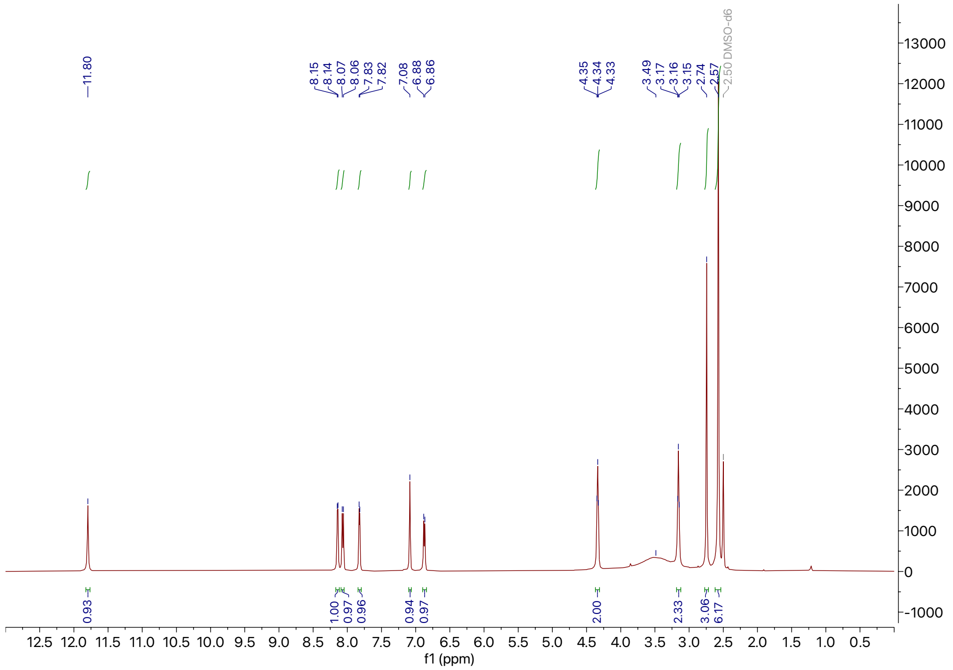

^{1}H NMR. 500 MHz, DMSO-d_6

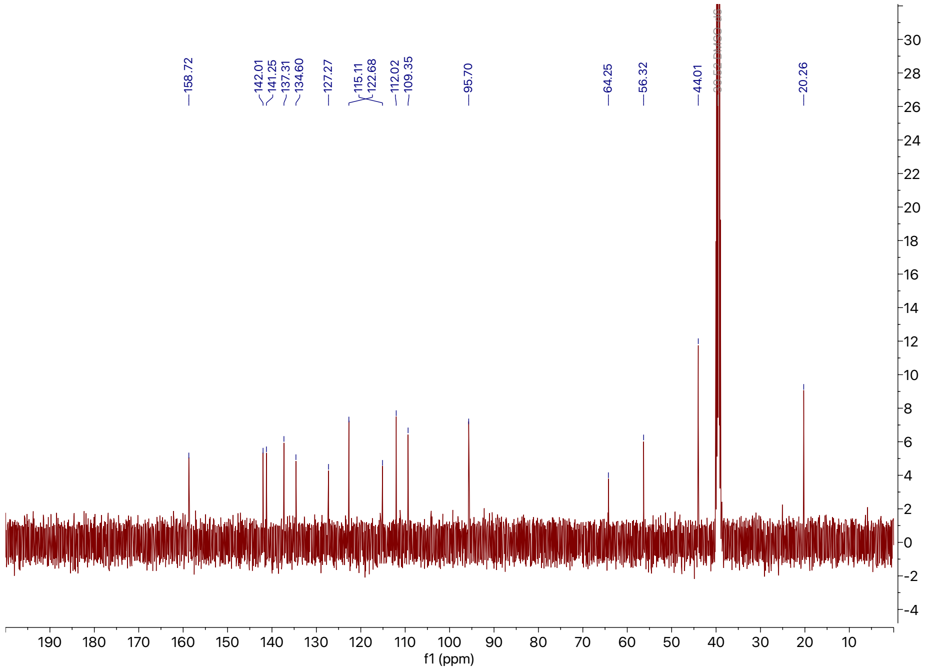

^{13}C NMR. 125 MHz, DMSO-d_6

9.

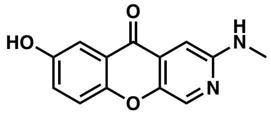

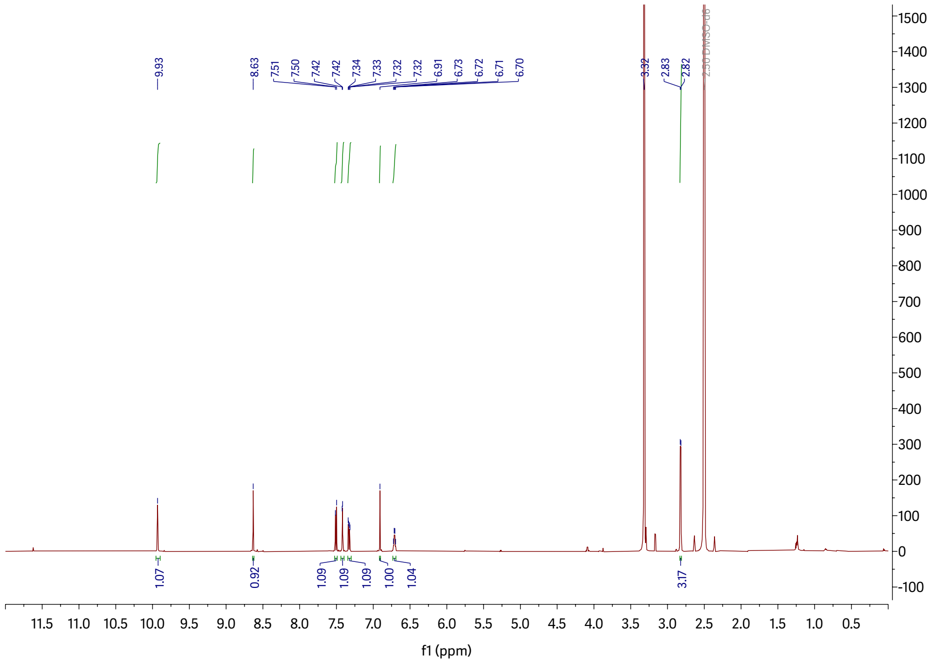

^{1}H NMR. 500 MHz, DMSO-d_6

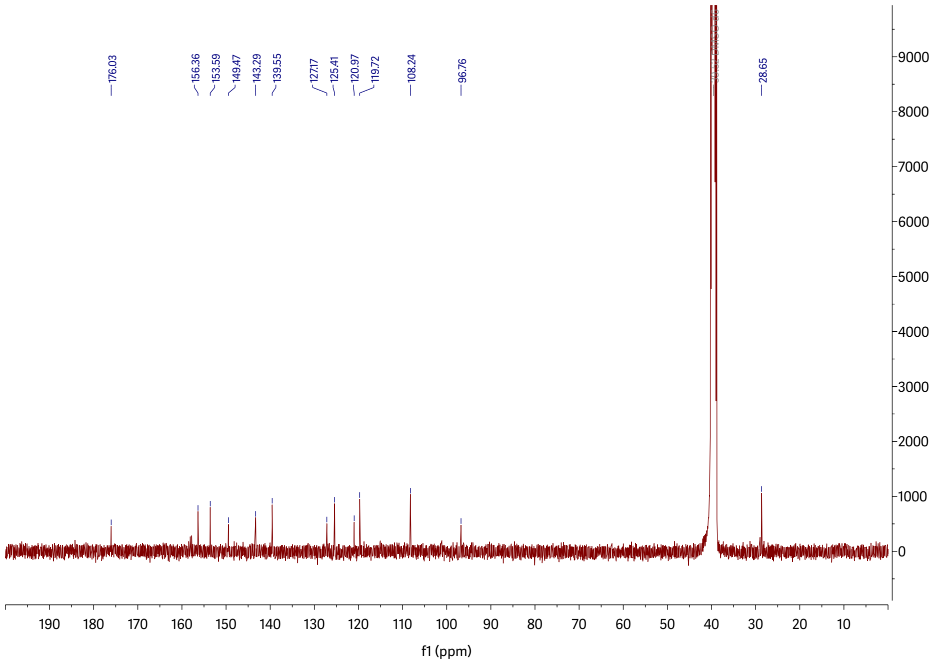

^{13}C NMR. 125 MHz, DMSO-d_6

SUPPLEMENTARY REFERENCES:

(1) Weiss, M. S. Global indicators of X-ray data quality. *Journal of Applied Crystallography* **2001**, *34*, 130-135. DOI: 10.1107/S0021889800018227FILE: /proj/ads/abstracts/.
(2) Karplus, P. A.; Diederichs, K. Linking Crystallographic Model and Data Quality. *Science* **2012**, *336* (6084), 1030-1033. DOI: doi:10.1126/science.1218231.
